# Ketogenic therapy towards precision medicine for brain diseases

**DOI:** 10.3389/fnut.2024.1266690

**Published:** 2024-02-21

**Authors:** Yang Liu, Linlin Fan, Haoying Yang, Danli Wang, Runhan Liu, Tikun Shan, Xue Xia

**Affiliations:** ^1^Translational Medicine Center, Huaihe Hospital of Henan University, Henan University, Kaifeng, China; ^2^Henan Key Laboratory of Brain Targeted Bio-nanomedicine, School of Life Sciences, Henan University, Kaifeng, China; ^3^Zhoushan People’s Hospital, Zhoushan, China; ^4^Neurosurgery Department, The First Affiliated Hospital of Zhengzhou University, Zhengzhou University, Zhengzhou, China; ^5^School of Environmental and Life Sciences, College of Engineering, Science and Environment, University of Newcastle, Callaghan, NSW, Australia

**Keywords:** ketogenic diet, precision medicine, brain disease, nutrigenomics, neural disease

## Abstract

Precision nutrition and nutrigenomics are emerging in the development of therapies for multiple diseases. The ketogenic diet (KD) is the most widely used clinical diet, providing high fat, low carbohydrate, and adequate protein. KD produces ketones and alters the metabolism of patients. Growing evidence suggests that KD has therapeutic effects in a wide range of neuronal diseases including epilepsy, neurodegeneration, cancer, and metabolic disorders. Although KD is considered to be a low-side-effect diet treatment, its therapeutic mechanism has not yet been fully elucidated. Also, its induced keto-response among different populations has not been elucidated. Understanding the ketone metabolism in health and disease is critical for the development of KD-associated therapeutics and synergistic therapy under any physiological background. Here, we review the current advances and known heterogeneity of the KD response and discuss the prospects for KD therapy from a precision nutrition perspective.

## 1 Introduction

**Figure 1:**
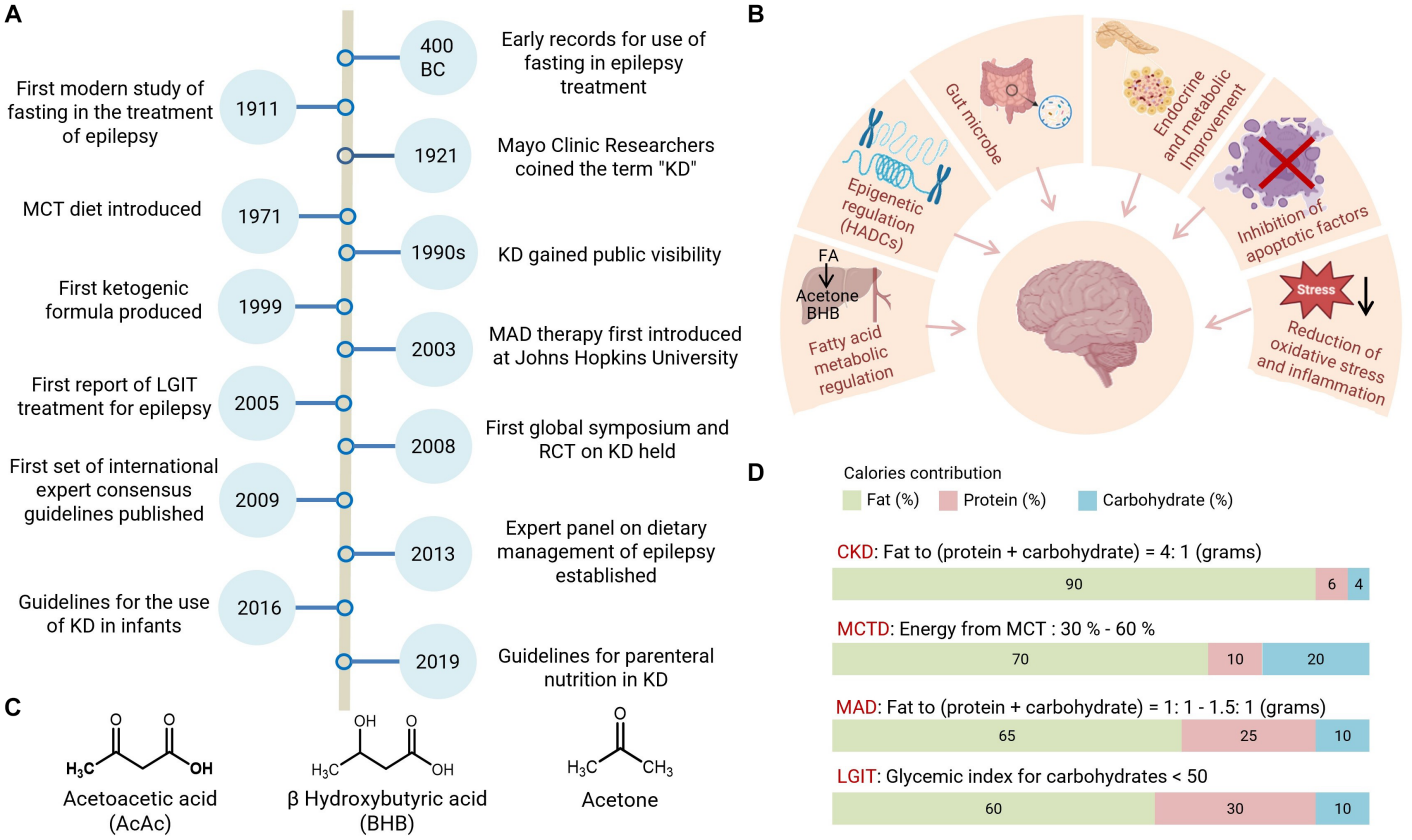
History and classification of the KD therapy. **(A)** Highlighted events in the development of ketogenic diet therapy. **(B)** Mechanistic hypothesis for KD treatments. **(C)** Structure of ketones, Acetoacetate (AcAc), β-hydroxybutyrate (BHB), and acetone. **(D)** Nutrient composition for the main KD variants. RCT, randomized clinical trial; CKD, classic ketogenic diet; MCTD, medium-chain triglyceride diet; HDACs, histone deacetylases.

The ketogenic diet (KD) is a high-fat diet that also restricts carbohydrates, and triggers the body’s production of ketones for energy use. The change in these macronutrient ratios from the original diet to KD rewires human energy metabolism to utilize ketones, which are derived from fatty acids, as an energy source instead of glucose (1). KD is one of the most widely used clinical diets for disease treatment and has become an established non-pharmacological intervention for many neurological diseases. As early as 1921, Drs. Rollin Turner Woodyatt and Russell Morse Wilder at the Mayo Clinic coined the term “ketogenic diet” and proposed that dietary ketones were as effective as fasting for the management of epilepsy but with long-lasting results (2). KD has therefore been intensively studied for the treatment of epilepsy. After the discovery of diphenylhydantoin as a chemical drug for seizures in 1938, the attention of physicians and researchers shifted from KD to the new anti-epileptic drug. At that time, interest in KD for the treatment of this disease decreased and did not come to people’s attention again until the 1990s (2, 3), when its therapeutic effects were gradually found to be effective in a wide range of diseases and more therapeutic potentials of KD were proposed. To clinically apply KD as a nutritional aid, standardization and specialization are extremely important. The “International Ketogenic Diet Expert Consensus Guidelines” were published in 2009 and have been frequently updated since then. Key milestones in KD therapy are summarized in Figure 1. In this review, our search strategy was designed by a multi-disciplinary team with backgrounds in nutrition, pharmacy, neurology, and clinical medicine; the goal was to ensure coverage of a wide range of issues and literature including and emphasizing the most classic publications screened by high citations or published by leading scholars. The most recent research was screened based on recent 5-year findings. We conducted an initial review via Google Scholar and PubMed and screened the available titles and abstracts. A further detailed review included highly relevant literature and cited references. Documents with less relevance were retrieved for further review.

## 2 Heterogeneity of KD therapy and the need for precision nutrition

The physiological function of KD is mainly triggered by ketones. Acetoacetate (AcAc), β-hydroxybutyrate (BHB), and acetone are collectively known as ketones or ketone bodies (4) (Figure 1), which can be generated from a series of biochemical reactions associated with fatty acid oxidation and are key mediators of KD therapy. Both BHB and AcAc are metabolizable and can be used as energy sources in different tissues. Ketogenesis is the term that describes the chemical reaction process that generates ketones. Fatty acid oxidation activated by starvation or diabetes triggers high levels of ketone production in the liver. This ketone-generating metabolic process is known as ketosis, which burns fat for fuel when the body lacks sufficient carbohydrates as an energy source.

To achieve greater flexibility and adherence to metabolic switch with KD, different formulations have been developed over the past decades, with four main KD variants now widely accepted by researchers and physicians: Classic KD (CKD), Modified Atkins Diet (MAD), Medium Chain Triglyceride Diet (MCT), and Low Glycemic Index Therapy (LGIT) (5). Classic KD is a rigid diet in which fat provides 90% of calories and its main source is Long-Chain Triglycerides (LCT) from food intake (1). Classic KD is widely used in clinical practice and is recommended for children under the age of two (5). For adolescents and adults, the majority (72%) of the consensus group prefers MAD or LGIT, mainly due to better adherence (5). MAD is a low-glycemic index (GI < 50) diet; it does not restrict calories and fluids due to its high-fat content and supplies more protein than classic KD. MAD is increasingly used in adults due to its better availability and tolerability (6). Its formulation features approximately 65% fat, 25% protein, and 10% carbohydrates (7). Another low-GI formula, LGIT, is based on a macronutrient composition of 60% fat, 30% protein, and 10% carbohydrates (7, 8). Studies have demonstrated that LIGT has a good seizure control effect and fewer side effects, which makes it an effective option for managing seizures associated with Angelman syndrome (9). However, LGIT is not recommended for the treatment of glucose transporter-1 deficiency syndrome (Glut1DS) or pyruvate dehydrogenase deficiency (PDHD) due to inadequate ketosis induction (5). Meanwhile MAD has shown good results in the treatment of Glut1DS (8). MCT is primarily composed of up to 60% medium-chain fatty acids from coconut or palm kernel oil used as a dietary supplement. MCT provides an option for people with carnitine deficiency, as carnitine is necessary for the processing of long-chain fatty acids provided by classic KD and it supplies more carbohydrates and protein for better compliance (10). It is also important to consider certain limitations when initiating MCT, as it may result in adverse effects such as diarrhea, nausea, and vomiting. Notably, the combination of MCT and valproic acid has been reported to be associated with liver failure; therefore, patients taking valproic acid should avoid MCT (11).

It is noteworthy that different types of KDs may have distinct effects on patient outcomes. A meta-analysis reviewed 12 studies that used CKD, MAD, and a combination of CKD and MCT for the treatment of anti-epileptic drug-resistant epilepsy in adults. Data from 270 patients showed that CKD had a response rate of 52%, while MAD had a response rate of 34%, suggesting that CKD may be more effective in some cases. However, compliance is lower with CKD (38%) compared to MAD (56%) in adult patients (12).

This evidence demonstrates wide variation in KD formula selections and corresponding treatment outcomes, which suggests that the selection of a specific KD treatment for patients should be based on the patient’s circumstances, and individual family genetic background, as well as the expertise guiding patient’s adherance. This is crucial to achieving optimized therapeutic efficacy and minimizing potential side effects. In addition, as a standard treatment, KD should be closely monitored and regulated to ensure its efficacy and promote dietary adherence. Currently, urine dipsticks (4), blood parameters, and breath sensors can be applied so as to monitor ketosis in clinics, and these points highlight the need for precision nutrition in clinical KD treatments.

## 3 Physiological benefits and therapeutic potential of the ketogenic diet in the nervous system

There is a consensus that an animal fat-centered diet contributed significantly to the evolution of a larger human brain (13). Hunting, rather than scavenging, allows humans to consume more energy-intense fatty meats, thus allowing more free energy to be redirected to fuel the brain (14). Ketosis may be the result of human evolution and may be strongly tied to a time when food scarcity occurred the majority of the time. The human body evolves to better utilize and conserve energy under such living conditions (15). Starvation results in global shifts in the body’s circulating metabolites. In particular, ketone bodies act as a substitute for glucose in providing energy to the brain. Specifically, the concentration of β-OHB can increase 13-fold when the human body is subjected to prolonged starvation. The level of ketones, which contribute to cerebral metabolism, also increases profoundly. The source of such ketone synthesis in the liver, when the body is under glucose starvation, is smart from the point of view that fatty acids themselves, originating from the body’s fat reserves, cannot cross the blood–brain barrier and thus satisfy the brain’s energy needs (16, 17).

### 3.1 Essential role of ketone metabolism in the neonatal brain

Compared to the adult brain, the brain of neonates utilizes ketones and lactate to a much greater extent than glucose (18). In neonatal rats, BHB and AcAc are preferred to glucose as they are substrates for the synthesis of phospholipids and sphingolipids, which are required for brain growth and myelination (19, 20). To date, the ketone body content of the human neonatal brain is reported to be 40-fold higher than that of the adult brain (21). Ketone bodies are a necessary energy source for fetal neurodevelopment, and monocarboxylate transporters are considered responsible for ketone bodies across the blood–brain barrier (22). Ketones are present in the blood of neonates and pregnant women, and even a few days after birth, human infants remain in ketosis (23). With the capacity to freely cross the placenta, exogenous ketone treatment has been reported to preserve cerebral energy metabolism, ameliorate brain damage, and display effective neuroprotective potency during neonatal hypoxia-ischemia (24, 25). Data also suggest that 5 mM BHB in the circulation may improve the survival rate of brain cells (26). In a rodent model of neonatal hypoxia, induction of ketosis was reported to reduce brain damage after exposure to 3 h of hypoxia (27). Collectively, this evidence suggests a protective role for ketone bodies in the neonatal brain under extreme conditions.

### 3.2 Benefits of a ketogenic diet in the adult brain

Ketones also contribute uniquely to the maturation of the nervous system by promoting myelin formation. Studies have indicated that KD is useful in adult remyelination, during which axon damage is repaired and attenuated (28). The neuronal protective effects induced by KD also involved multiple pathways, such as modulation of ATP-sensitive potassium (K-ATP) channels, enhanced purinergic and GABAergic neurotransmission, increased expression of brain-derived neurotrophic factor (BDNF), attenuation of neuroinflammation, expansion of bioenergetic reserves, and stabilization of the neuronal membrane potential through improved mitochondrial function (29). The benefits associated with KD have been extensively reviewed (30, 31). As the key functional KD factor, BHB directly regulates inflammation and neurotrophic factors by inhibiting the activation of the innate immune sensor NLRP3 and inhibiting HDAC, thereby maintaining, restoring, and improving brain function (32). Studies also demonstrate that ketones, which are biomarkers of the brain’s aging response to fuel sources, increase the stability of brain networks, while decreasing glucose, affecting overall brain activity (33).

### 3.3 The potential of a ketogenic diet to improve motor neuron function

In the peripheral nervous system, mice fed a KD diet show increased epidermal axon density and neurite outgrowth in sensory neurons (34), which implies that ketones may provide benefits to peripheral axons and sensory functional recovery. In an amyotrophic lateral sclerosis (ALS) mouse model (SOD1^G93A^), the administration of KD was able to promote ATP synthesis by bypassing the inhibited complex I in the mitochondrial respiratory chain, increasing mitochondrial energy production and membrane stabilization, leading to increased motor neuron survival and improved motor function compared to normally fed mice. Although human trials are still insufficient and the survival time of treated mice was not increased, researchers found both histological and functional improvement in the KD-treated ALS animal model, with higher motor neuron counts and less motor function impairment (35, 36). In line with this, motor function also showed improvement in rodent models on KD and/or MCT diets in neurodegenerative diseases and spinal cord injury (37, 38). Taken together, KD shows a broad spectrum of benefits to nervous system function through a variety of mechanisms.

## 4 Hypothetical functional mechanisms for ketogenic diet therapy

During diet-induced glucose deficiency, ketone bodies are generated in the liver and circulated in the bloodstream to metabolically active tissues for processing as energy substrates. In particular, BHB, in addition to its role as an energy source, also has a variety of molecular signaling and regulatory functions in a wide range of human diseases (39). BHB is considered to be the main efficacy factor in KD treatment. In addition to influencing the process of oxidative metabolism, BHB also plays a direct role as an endogenous ligand of the hydroxycarboxylic acid receptor 2 (HCA2), whose activation by nutritional or pharmacological approaches could ameliorate neuroinflammation (40). Moreover, ketone bodies are also important in the synaptic vesicular cycle (41), KD can also affect neurotransmitter levels in the synaptic cleft and increase GABA levels in addition to altering glutamate metabolism (42). BHB may well play its neuroprotective role through the above mechanisms.

### 4.1 Epigenetic regulation

BHB has been reported as a Class I histone deacetylase (HDAC) inhibitor and an endogenous neuroprotective epigenetic modifier (43). As an HDAC inhibitor, BHB is involved in histone regulation through acetylation. Through local histone acetylation induction at the promoter of oxidative stress resistance genes such as forkhead box O3 (Foxo3a) and metallothionein2 (Mt2) (44), BHB has bridged the connection between metabolic and epigenetic modifications and gene expression. Studies have also shown a more direct epigenetic effect of BHB, defining a novel histone modification as β-hydroxybutyrylation at H3K9 (45). KD can also reportedly increase the levels of lysine acetylation and p53 acetylation, which are some of the most important cell cycle regulators and tumor suppressors (46).

### 4.2 Gut microbiome

Gut microbes are also known to be involved in the development of many diseases. The dietary influence of KD on gut microbes is easily presumed. One study reported that KD significantly reduced the diversity of the intestinal flora, but at the same time, *Parabacteroides* and *Akkermansia muciniphila* increased during KD intake (47). Researchers found that a shift in the microbiome led to changes in the intestinal metabolome of the colon, resulting in a decrease in gamma-glutamyl amino acids, which may be beneficial for seizure protection (48). The influence of the gut microbiome on disease is still largely unknown, and the underlying mechanism requires further investigation. Moreover, significant changes in microbial communities combined with different dietary compositions may drive different molecular inputs to neuroendocrine signaling pathways, and these altered inputs (e.g., short-chain fatty acid response) combined with changes in the gut-brain axis may drive different systemic responses at the level of hormones and gene expression (49).

### 4.3 Oxidative stress and inflammatory pathways

Disturbances in mitochondrial energy metabolism and reactive oxygen species (ROS) production are key players in many diseases. KD significantly affects mitochondrial energy metabolism. For instance, studies have shown that KD increases the antioxidant glutathione levels, and activates the transcription factor Nrf2 (nuclear factor erythroid-2 related factor) detoxification pathway (50, 51). Both glutathione and Nrf2 are crucial for balancing ROS levels (52). Furthermore, in a glioma mouse model, KD induces anti-tumor effects and reduces ROS production in tumor cells by regulating ROS-associated gene expression (53).

Moreover, KD showed a powerful anti-inflammatory effect. Ketones, especially BHB, have been reported to reduce cardiac inflammation, decrease the development of heart failure (54), and ameliorate the inflammation after spinal cord injury (55). KD also has a promising role in inhibiting NOD-like receptor protein 3 (NLRP3) inflammasome assembly, reducing oxidative damage, and attenuating inflammatory mediators produced by infiltrating macrophages (56). Although evidence has been found and several hypotheses have been proposed, the clear mechanism for the benefit of KD still needs to be further explored.

## 5 Ketogenic therapy for precision medicine

### 5.1 Public need for precision nutrition and nutrigenomics

Precision medicine is a term that describes new therapeutic strategies with more precise targeting, focusing on subgroups of diseases with individual backgrounds, and is the new trend for a broad spectrum of disease treatment (57). The ultimate aim of precision medicine is to maximize the outcome by individualizing a broad range of patient features, including genome profiling, drug response, disease development period, physiological states, and causal inference. Formalized precision medicine treatment consists of a series of decision-making points with recommended actions, such as drug and dose selection, timing of administration, specific dietary or exercise recommendations, and other aspects of treatment that can be made following up-to-date patient information (58).

**Figure 2:**
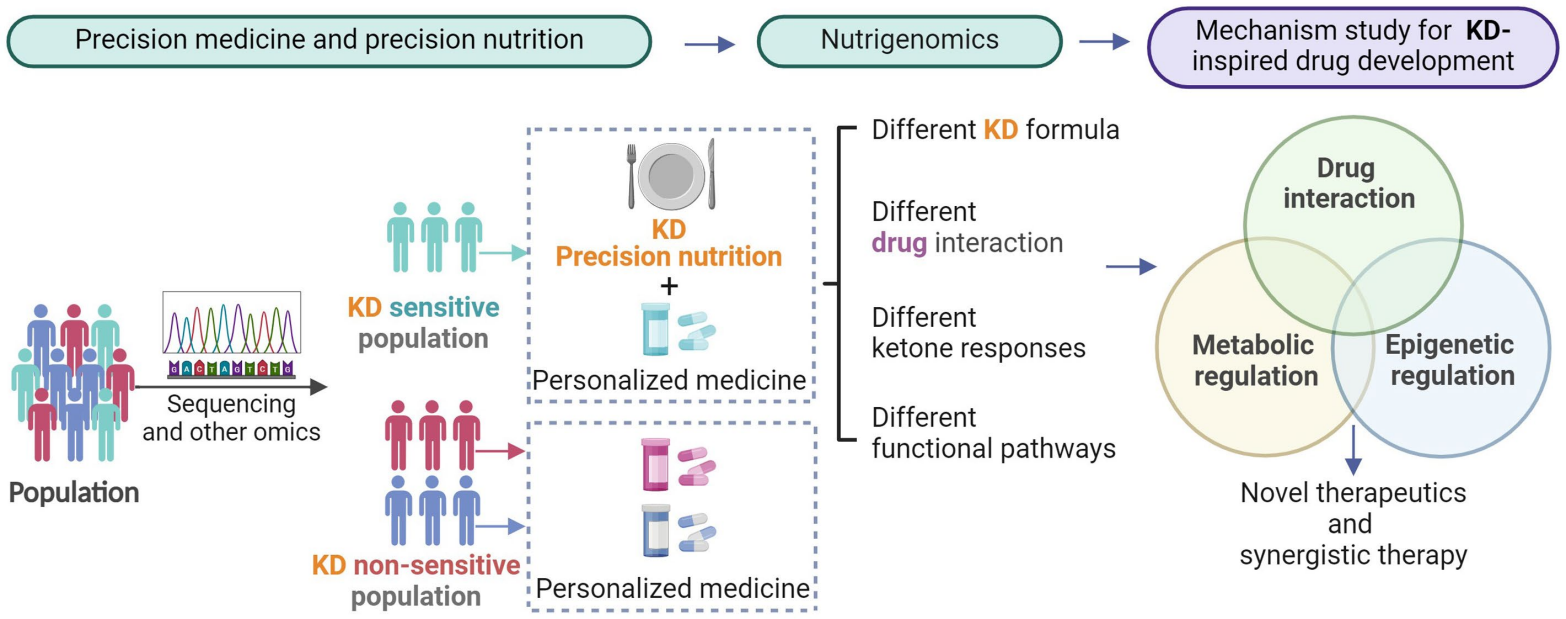
The need for precision medicine and precision nutrition in disease therapy. The emerging understanding of precision nutrition and nutrigenomics enables a more focused study of ketone-triggered metabolic and epigenetic crosstalk and keto-drug interactions.

Despite clear evidence of the impact of nutrients on health, diet as an environmental exposure is not fully highlighted in clinical practice and research. Recently, there has been an emerging awareness of “precision nutrition,” and more pieces of evidence have concluded that nutrient intake affects therapeutic outcomes (59). Thus more precision nutrition is being proposed to replace or augment drugs to some extent (60). The importance of nutrition to individual health makes nutrigenomics emerge as a hot discipline. Nutrigenomics investigates the impact of diet/nutrition on gene expression at the epigenomic, transcriptomic, proteomic, metabolomic, and microbiome levels (61). Conversely, the discipline of nutrigenomics is the study of how genes affect the body’s response to food (62). All of these new disciplines aim to clarify the interaction of health, genes, and diet. Heterogeneity in patient-made precision nutrition and nutrigenomics is extremely important in KD therapy (Figure 2).

### 5.2 The ketogenic diet is beneficial for neuronal diseases and could be greatly influenced by precision medicine

KD has been recommended for the treatment of certain epileptic and genetic syndromes including Dravet syndrome (63), myoclonic-atonic seizures (Doose syndrome) (64), West syndrome (infantile spasms) (65), febrile infection-related epilepsy syndrome (FIRES) (66), Ohtahara syndrome (67), and super-refractory focal and myoclonic status epilepticus (68). Case reports have also indicated beneficial effects of KD in epileptic encephalopathies such as Lafora body disease (69), early infantile epileptic encephalopathy (70), Landau–Kleffner syndrome (71), subacute sclerosing panencephalitis (72), adenylosuccinate lyase deficiency (73), juvenile myoclonic epilepsy (74), CDKL5 encephalopathy (75), infantile epilepsy with migrating focal seizures (76), childhood absence epilepsy (77), and epileptic encephalopathy with continuous spike-and-wave during sleep (78). Metabolic-to-epigenetic modulation is considered to be one of the main reasons for the therapeutic effect of KD in neurodevelopmental disorders. Although KD has shown therapeutic potential in these diseases, heterogeneity has shown varied results in KD therapy for specific disease subtypes, the clear mechanisms of which are still largely unknown. Some examples of KD therapeutic effects and exceptions are summarized below.

*Epilepsy* is a common neurological disorder affecting more than 70 million people worldwide with genetic mutation as one of the leading causes (79). Dietary therapy for epilepsy has a long history and was recorded in the Hippocratic Collection (7, 80). KD has now become an important alternative treatment for patients with refractory epilepsy, both in children and adults (81–83). Numerous evidence have shown control of epilepsy incidence, but its cure is still extremely challenging as many refractory epilepsies are caused by genetic mutations. Some studies also concluded the failure of KD therapy. For example, patients with epilepsy with GLUT1 deficiency syndrome show non-sensitivity to KD despite adequate ketosis (84). The genetic background of patients also showed differences in keto response and keto-drug interaction, making precision medicine even more important (85, 86).

*Kabuki syndrome* is an intellectual disability caused by mutations in either the *KMT2D* or *KDM6A* genes, which are involved in histone acetylation and chromatin accessibility (87). In the Kmt2d*^+/βGeo^* mouse model, treatment with KD for 2 weeks normalizes the global histone modification state corrects the neurogenesis deficiency in the granule cell layer of the dentate gyrus, and rescues the hippocampal memory defects (88). These could also be achieved by administering exogenous BHB (89). The mechanism behind this may be due to improved chromatin accessibility and the transcriptional regulatory network.

*Rett syndrome* is most often caused by a mutation in the key epigenetic regulator gene that encodes the methyl-CpG binding protein. This mutation leads to seizures and intellectual disability (90). Patients with Rett syndrome treated with KD show reduced seizures and great improvements in social and behavioral functioning (91).

*Angelman syndrome* is a genomic imprinting disorder associated with multiple anomalies and intellectual disability. In a mouse model of Angelman syndrome, dietary supplementation with ketones has been shown to significantly reduce the frequency of seizures and improve overall neural function including behavior, learning, and memory (92). Clinical trials in children with Angelman syndrome are currently testing ketone supplementation for seizure control (93).

*Psychiatric disorders and comorbidity with epilepsy*. KD has also been proven to be useful in the treatment of psychiatric disorders in comorbidity with epilepsy in children and adolescents (94), as well as in psychiatric disorders such as anxiety disorder (95), bipolar disorder (96), schizophrenia (97), depression (98), autism spectrum disorder and attention-deficit/hyperactivity disorder (ADHD) (99). The positive outcome may be attributed to the profound impact of ketones on multiple targets, including but not limited to glutamate/GABA transmission, monoamine levels, mitochondrial function and biogenesis, neurotrophism, oxidative stress, insulin dysfunction, and inflammation, and demonstrates mood-stabilizing and antidepressant effects (100).

*Alzheimer’s disease* (*AD*) is the most common neurodegenerative disease and the leading cause of dementia in the elderly population (101). The brains of AD patients show decreased glucose uptake (102). As a backup fuel for the brain, ketone bodies can cross the blood–brain barrier, thereby improving the function of energy-starved neurons. It has been proven that KD intervention results in increased ketone utilization and brain network stabilization, which could be beneficial for the aging brain (33). The mechanism behind this may be that KD alleviates energy starvation and neurotransmitter imbalance in the AD brain, as the brain metabolism of ketones can support up to 30% of glutamate and glutamine carbon (48) through the glutamate decarboxylase reaction (103). In animal models, 8 months of KD feeding in middle-aged mice (8.5 months old) improved cognition and ameliorated Aβ and tau pathology (104). In the 3xTg mouse model of AD, ketone metabolism can restore TCA cycle metabolites, thereby enhancing amino acid biosynthesis and contributing to behavioral performance (105). Increased ketone levels also reduce the brain’s usage of glucose; as ketones are used preferentially over glucose by brain cells, Alzheimer’s brains are even more efficient at taking up ketones (106). KD has also shown the potential to prevent AD in high-risk populations (107, 108). AC-1202, an oral ketogenic compound that can induce a mild state of ketosis without modifying normal diets, was developed to improve cognitive performance and resulted in significant differences in the AD Assessment Scale-Cognitive subscale scores compared to the placebo (109).

Exceptions: It should be noted that KD shows variable outcomes for different genetic backgrounds. In this case, the oral ketogenic agent AC-1202 resulted in a statistically significant improvement in cognitive performance in patients who were *APOE4* negative, but not *APOE4* positive (109). In this case, precision nutrition is extremely important in the treatment of the disease.

*Parkinson’s disease* also benefits from KD (110). In animal models, 1-methyl-4-phenol-1,2,5,6-tetrahydropyridine (MPTP) is used to induce destruction of dopaminergic neurons in the substantia nigra, mimicking human Parkinson’s-like syndrome (111), while the infusion of BHB was found to have a protective role in dopaminergic neurodegeneration and motor deficits induced by MPTP (112). Ketones also protect substantia nigra dopaminergic neurons against 6-hydroxydopamine neurotoxicity in a rat model of Parkinson’s disease (113). In Parkinson’s disease patients, studies have revealed that high ketones are associated with improvements in Unified Parkinson’s Disease Rating Scale (UPDRS) scores (114).

*Brain cancer* cells showed significant metabolic alterations with increased glucose and hydroperoxide metabolism compared to normal cells (115). There is evidence that cancer cells are intolerant to ketones both *in vitro* and *in vivo* (116). Preclinical studies have shown promising results for KD in reducing tumor growth and extending survival in the brain (117), breast (118), prostate (119), and gastric cancer models (120).

Exceptions: Despite KD showing a general benefit in many diseases, KD is not suitable for all diseases and even the formula of KD varies in different cases, raising the need for precision nutrition and nutrigenomic research.

For instance, not all cancer subtypes are amenable to KD. Some studies showed the absence of positive effects on tumor progression and survival with a 3:1 ratio of fatty acids to carbohydrates in a KD regimen in glioblastoma mouse models but changed to positive when the ratio increased to 4:1 or 6:1 (117). On the contrary, in medulloblastoma mouse models, no change in survival or tumor progression was found even when the ratio of fatty acids to carbohydrates ratio was increased to 4:1 or 6:1 (117). This also highlighted the need for precision nutrition in clinical practice.

### 5.3 Concerns raised for ketogenic therapy call for personalized medicine

Notably, KD is used as a standard therapy for specific diseases and is not recommended for daily use by healthy people. Some case reports have even raised concerns about long-term KD use and under the background of some diseases. Despite its efficacy in the treatment of epilepsy from different causes, the benefits of KD rely on the integrity of the ketone body synthesis pathway. Inborn genetic errors in lipid metabolism such as membrane long-chain fatty acid transport, β-oxidation, and the Krebs cycle could be fatal when implementing fasting or KD (121). Researchers highlighted that carnitine deficiency, carnitine palmitoyl-transferase I or II deficiency, carnitine translocase deficiency (122), β-oxidation defects (123), pyruvate carboxylase deficiency (124) should be screened before KD treatments. Also, KD is not entirely free of side effects, with fatigue, muscle cramps, hypotension, constipation, and unwanted weight loss being the most commonly reported (125). Although KD has been used by healthy people for weight loss, severe obesity is often driven or accompanied by a variety of metabolic disorders. The crosstalk for different metabolic pathological conditions should be emphasized, and fortunately the safety of long-term KD for weight loss is gradually being noticed (49, 126). Thus, though KD is generally considered a safe treatment, there are limitations in current KD treatments and one should always weigh its long-term pros and cons before initiation (127). A summary of concerns regarding KD treatment is provided below.

- Contraindications: liver failure, pancreatitis, inborn disorders of fat metabolism, primary carnitine deficiency, carnitine palmitoyltransferase deficiency, carnitine translocase deficiency, porphyria, and pyruvate kinase deficiency (127).
- Potential long-term side effects: hepatic steatosis, kidney stones, hypoproteinemia, and vitamin deficiency (127).
- Potential short-term side effects: keto breathiness and “keto flu,” which include fatigue, headache, dizziness, nausea, vomiting, constipation, low exercise tolerance, etc. (127).
- Incorrect medication dosing may cause severe hypoglycemia in diabetic patients (127).
- May lead to ketonemia and ketonuria (127).
- An increase in low-density lipoprotein cholesterol (LDL-C) levels may lead to atherosclerosis acceleration and increased CVD risk (127).
- More solid evaluation required in clinical trials: increased sample sizes, duration of interventions, decreased participant dropout rates, and follow-up for long-term response (127).
- Women with gestational diabetes mellitus are advised to avoid a ketone-elevating diet (128).
- KD is a lifelong treatment for some diseases, and therefore relatively low compliance rates (38% for CKD, 56% for MAD) were reported in a meta-analysis of 270 patients (12).
- The genomic variations between individuals may mean that the risks of KD may outweigh the benefits.

## 6 Conclusion

As an endogenous fuel that bridges metabolism to epigenetic regulation, the recent understanding of the function of ketones in both areas is encouraging for the development of new drugs for these previously identified “incurable diseases.” The benefits of ketogenic intervention in clinical practice are thusly highlighting its therapeutic potential for multiple brain diseases. Ketogenic intervention can be simply achieved through an associated diet that is relatively affordable and accessible. Growing evidence suggests that KD has a therapeutic effect on a wide range of human diseases, such potential makes KD an encouraging therapeutic strategy for many brain diseases. Being a low-side-effect treatment, elucidation of its therapeutic mechanism is a promising topic in the development of novel biotechnological drugs and synergistic therapy. However, the mechanism of KD benefits is still not fully understood, despite some evidence indicating its multiple targets and ability to regulate histone deacetylase, glycolysis, neurotransmitter levels, the function of NLRP3 inflammasome, and oxidative stress.

Moreover, KD is not suitable for all diseases and even the ratio difference in the KD formula may lead to unexpected therapeutic outcomes, thus increasing the need for precision nutrition and nutrigenomics research. A recent study revealed a distinct cell type–specific KD response in the brain: metabolic plasticity is found in astrocytes and neurons, but not in oligodendrocytes (129), which highlights the importance of narrowing the variation in KD responses. Available standards for clinical KD therapies are still very limited, and a certain level of precision nutrition is required as KD moves into clinical translation. Further studies to identify the clear mechanisms of KD and drug interactions, different KD responses in heterogeneous patient backgrounds, and clear mechanisms of KD intervention in the fight against numerous specific central nervous system diseases are crucial. In addition, in clinical applications, precision nutrition should also be considered as slight changes in dietary composition could impact/ameliorate the potential side effects of KD in certain populations.

## 7 Open questions

1. From an evolutionary point of view, how do we understand the physiological benefits of KD compared with a normal diet?
2. How do we define the drug interactions with KD in different individuals during the diagnosis and early treatment period?
3. Is it possible to trigger ketone production on a cell-specific or tissue-specific level?
4. Can KD regulate the immune system? Will combination therapies such as chemotherapy, immunotherapy, and KD improve the survival of cancer patients?
5. Can KD be a trigger for synergistic drug treatment?
6. What is the epigenetic map of patients following a KD regimen? Is there any drug or epigenetic regulator that can replace long-term KD administration?

## Author contributions

YL: Writing – original draft, Writing – review & editing, Conceptualization, Validation, Visualization. LF: Investigation, Visualization, Writing – review & editing. HY: Formal analysis, Validation, Visualization, Writing – review & editing. DW: Conceptualization, Validation, Writing – original draft, Writing – review & editing. RL: Validation, Visualization, Writing – review & editing. TS: Conceptualization, Supervision, Validation, Writing – original draft, Writing – review & editing. XX: Conceptualization, Formal analysis, Supervision, Validation, Visualization, Writing – original draft, Writing – review & editing.
